# Recent dermatophyte divergence revealed by comparative and phylogenetic analysis of mitochondrial genomes

**DOI:** 10.1186/1471-2164-10-238

**Published:** 2009-05-21

**Authors:** Yuan Wu, Jian Yang, Fan Yang, Tao Liu, Wenchuan Leng, Yonglie Chu, Qi Jin

**Affiliations:** 1 Department of Microbiology and Immunology, Medical School of Xi'an Jiaotong University, Shaanxi, 710061, PR China; 2 State Key Laboratory for Molecular Virology and Genetic Engineering, Institute of Pathogen Biology, Chinese Academy of Medical Sciences, Beijing 100730, PR China

## Abstract

**Background:**

Dermatophytes are fungi that cause superficial infections of the skin, hair, and nails. They are the most common agents of fungal infections worldwide. Dermatophytic fungi constitute three genera, *Trichophyton*, *Epidermophyton*, and *Microsporum*, and the evolutionary relationships between these genera are epidemiologically important. Mitochondria are considered to be of monophyletic origin and mitochondrial sequences offer many advantages for phylogenetic studies. However, only one complete dermatophyte mitochondrial genome (*E. floccosum*) has previously been determined.

**Results:**

The complete mitochondrial DNA sequences of five dermatophyte species, *T. rubrum *(26,985 bp), *T. mentagrophytes *(24,297 bp), *T. ajelloi *(28,530 bp), *M. canis *(23,943 bp) and *M. nanum *(24,105 bp) were determined. These were compared to the *E. floccosum *sequence. Mitochondrial genomes of all 6 species were found to harbor the same set of genes arranged identical order indicating that these dermatophytes are closely related. Genome size differences were largely due to variable lengths of non-coding intergenic regions and the presence/absence of introns. Phylogenetic analyses based on complete mitochondrial genomes reveals that the divergence of the dermatophyte clade was later than of other groups of pathogenic fungi.

**Conclusion:**

This is the first systematic comparative genomic study on dermatophytes, a highly conserved and recently-diverged lineage of ascomycota fungi. The data reported here provide a basis for further exploration of interrelationships between dermatophytes and will contribute to the study of mitochondrial evolution in higher fungi.

## Background

Dermatophytes are parasitic fungi that infect skin, hair and nails of both humans and animals. They are the primary causative agents of dermatophytosis, a major public health concern in some geographic regions[1-3]. While not fatal, dermatophyte infections cause significant morbidity and are of significant cost to society because of their chronic nature and resistance to therapy. Dermatophytes encompass 3 genera, *Trichophyton*, *Epidermophyton*, and *Microsporum*. *T. rubrum*, *T. mentagrophytes *and *M. canis *are the most common species in hospital isolates (72–95%)[4,5]. Morbidity is less commonly associated with *M. nanum *while *T. ajelloi *is a geophilic fungus that only rarely infects human.

Mitochondria are generally accepted as descendants of endosymbiotic alpha-proteobacteria[6,7] and are considered to be of monophyletic origin[8-10]. As vital physiological processes and basic adaptive strategies do not always correlate with trees derived from ribosomal sequences[11,12], mitochondrial DNA (mtDNA) sequences have become a popular tool for phylogenetic studies. Individual gene sequences often contain a limited number of informative sites and can lead to incongruent phylogenetic trees. In contrast, entire mitochondrial genomes tend to produce reliable phylogenetic trees[11-13]. Despite the emergence of new technologies for rapid DNA sequence determination, sequencing of complete mtDNAs is still more feasible and economical than whole-genome sequencing. Furthermore, complete mtDNA sequences reveal gene content, order and position, and provide further information regarding introns and intergenic regions [10].

The number of mitochondrial genomes sequenced has increased greatly over the past decade, notably through the interdisciplinary collaboration of the Organelle Genome Megasequencing Program . Some thousands of complete mtDNA sequences are already available from taxonomically diverse organisms including fungi, plants and animals. This resource provides an unprecedented insights into the origin and evolution of the mitochondrial genome [13]. In comparison to the genomes of free-living alpha-proteobacteria, the number of genes contained within the modern mitochondrial genome has been greatly reduced. It is inferred that many previously functional genes have been transferred to the nucleus; others appear to have been replaced by pre-existing nuclear genes of similar function [14]. Moreover, recent studies have suggested that positive selection plays a role in mitochondrial evolution [15-17] while mtDNA polymorphisms are thought to be maintained within populations via selection on the joint mitochondrial-nuclear genotype [15].

The fungal mitochondrial genome typically contains 14 conserved protein-coding genes, 22–26 tRNA genes, and 2 rRNA genes[9,18-20]. mtDNA divergence between different fungal species is predominantly associated with variation in intergenic regions, intronic sequences, and gene order[13,21]. The Fungal Mitochondrial Genome Project was launched over a decade ago [22] and more than 50 complete mitochondrial genomes of fungi have been determined to date (available from GenBank website ). Nevertheless, only one sequence is derived from a dermatophyte, i.e. *E. floccosum *[12]. Indeed, the dearth of publicly available genomic data is a major barrier to biomedical research on dermatophytes [23].

We report here complete mtDNA sequences for 5 dermatophytes including 3 species of *Trichophyton *(*T. rubrum*, *T. mentagrophytes *and *T. ajelloi*) and 2 species of *Microsporum *(*M. canis *and *M. nanum*). These sequences, with the previously reported *E. floccosum *mtDNA sequence [12], have permitted systematic comparative analysis of dermatophytes. The mitochondrial genomes of dermatophytes are highly conserved, indicating that these superficial fungi are closely related. Furthermore, phylogenetic analysis based on complete mitochondrial genomes has revealed that dermatophytes arose very late among the ascomycota fungi. This is the first comparative genomic study on dermatophytes and will provide valuable insights into the genomics and phylogeny of this important group of fungal pathogens.

## Results and discussion

### General features

**Figure 1:**
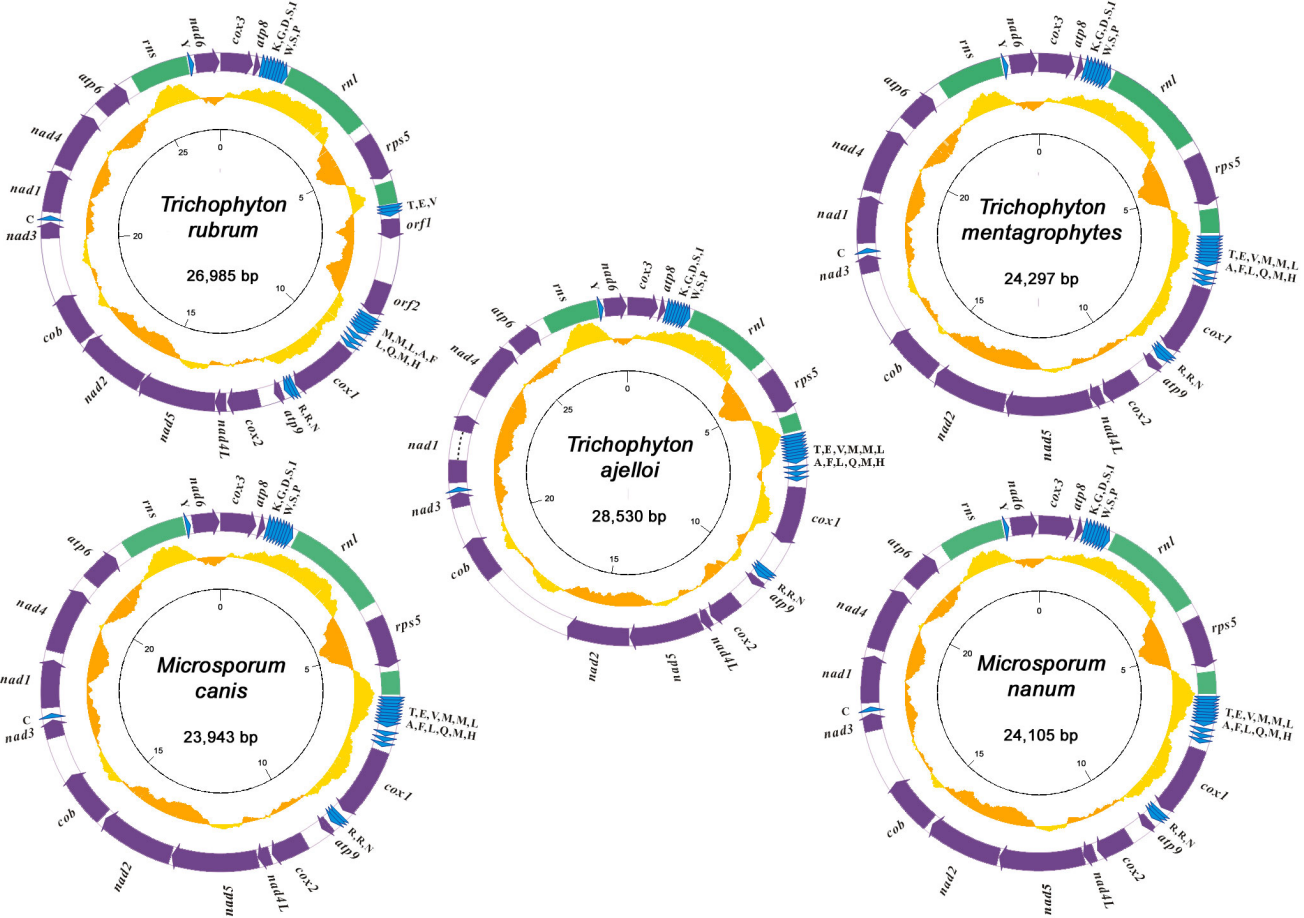
**Circular representations of the dermatophyte mitochondrial genomes**. Protein-coding genes are represented by purple arrows. rRNA and tRNA genes are indicated by green blocks and blue triangles respectively. G+C contents with a window size of 1 kb are shown in yellow (higher than genome average) or orange (lower than average) curves. The inner scale is marked at 5 kb intervals.

The mitochondrial sequences of *T. rubrum*, *T. mentagrophytes*, *T. ajelloi*, *M. canis *and *M. nanum *are circular DNAs of 26,985 bp, 24,297 bp, 28,530 bp, 23,943 bp and 24,105 bp, respectively (Fig. 1). The mitochondrial genome of *M. canis *is the smallest of known dermatophytes, while the previously determined *E. floccosum *is the largest and exceeds 30 kb [12]. The size differences between mitochondrial genome sizes are primarily due to interspecific variation in intergenic regions and introns (see below).

**Figure 2:**
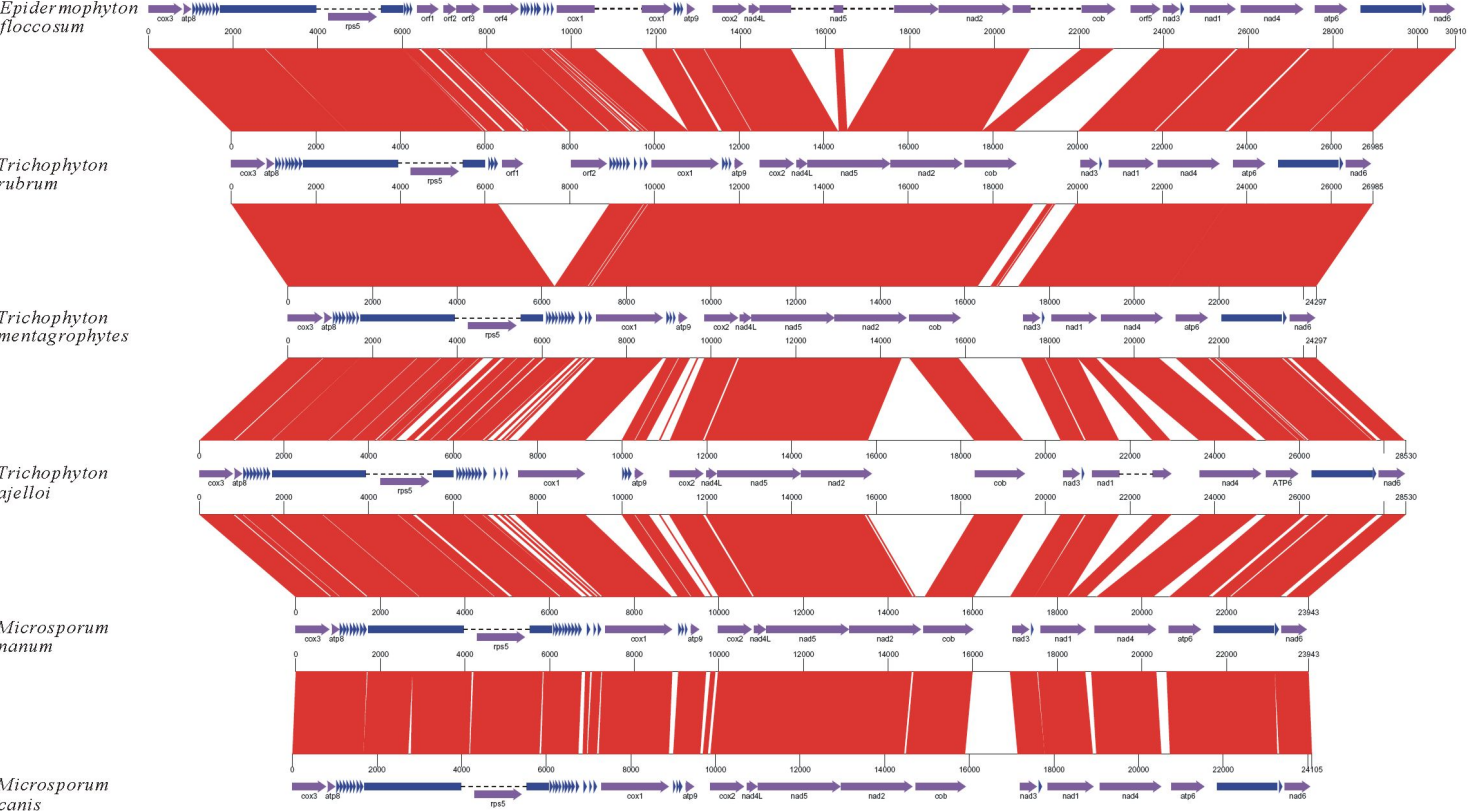
**Pairwise linear genome comparisons of dermatophyte mtDNAs**. Protein-coding genes are represented by purple arrows. rRNA and tRNA genes are shown by blue blocks and triangles respectively. Red parallelograms indicate the locations of homologous genomic regions in adjacent genome pairs.

With the exception of *T. rubrum *and *E. floccosum *that carry several additional hypothetical genes, each genome encodes 15 conserved protein-coding genes, 2 rRNA genes (*rnl *and *rns*), and 25 tRNA genes (Table 1, Fig. 1). The dermatophyte mitochondrial genomes are highly compact: structural genes account for >70% of each genome (Table 1). All genes are encoded on the same DNA strand. Moreover, all the mitochondrial genomes retain colinearity at the nucleotide level without detectable DNA rearrangement (Fig. 2). The high conservation of genome structure indicates that these dermatophytes are closely related.

**Table 1:** General features of the mitochondrial genomes of dermatophytes

**Species**	***T. rubrum***	***T. mentagrophytes***	***T. ajelloi***	***M. canis***	***M. nanum***	E. floccosum*
Genome size (bp)	26,985	24,297	28,530	23,943	24,105	30,910

G+C content (%)	23.51	24.03	23.45	24.15	24.47	23.43

No. of protein-coding genes	17	15	15	15	15	24

G G+C content of protein-coding genes (%)	22.17	22.30	22.38	22.79	22.94	22.00

No. of rRNAs/tRNAs	2/25	2/25	2/25	2/25	2/25	2/25

G+C content of RNA genes (%)	32.64	32.65	32.85	32.61	32.55	32.88

Percentage of structural genes (%)	79.72	83.01	70.34	84.09	83.76	87.37

No. of introns	1	1	2	1	1	5

No. of intronic ORFs	1	1	1	1	1	5

*Data from GenBank record AY916130.

The overall G+C content of the 5 genomes is ~24% (Table 1) consistent with the characteristic AT-rich nature of fungal mitochondrial genomes. The G+C content of genomic regions encoding RNA genes is usually higher than the genome average (Table 1) while the majority of protein-coding genes, with exception of *cox1*, have a lower G+C content (Fig. 1). The unusual G+C content of *cox1 *may reflect its unusual location sandwiched between 2 tRNA gene clusters.

### Protein-coding genes and rRNAs

The five mitochondrial genomes share 15 protein-coding genes. These include ATP-synthase subunits 6, 8, and 9 (*atp6*, *atp8*, and *atp9*), cytochrome oxidase subunits I, II, and III (*cox1*, *cox2*, and *cox3*), apocytochrome b (*cob*), a ribosomal protein (*rps5*), and NADH dehydrogenase subunits (*nad1*, *nad2*, *nad3*, *nad4*, *nad4L*, *nad5*, and *nad6*). *T. rubrum *carries 2 additional hypothetical genes (*orf1 *and *orf2*) located between *tRNA-Val *and *tRNA-Met *(Fig. 1). Both genes have homologs of >90% nucleotide similarity in the previously determined *E. floccosum *mitochondrial genome (GenBank: AY916130). The function of these putative genes is not yet known; further studies will be required to determine whether they play a role in mitochondrial function.

Synonymous base substitutions (dS) are considered to be selectively neutral while substitutions causing amino acid substitution, or non-synonymous substitutions (dN), are almost always adaptive mutations. The dN/dS ratio therefore affords an index of selective pressures operating on protein-coding genes. Because selection for adaptive amino acid substitutions increases dN, a dN/dS ratio of >1 is found in genes subject to selection for change (positive selection). In contrast, a dN/dS ratio of <1 indicates conservation of essential amino acid sequences (negative selection). We calculated the dN/dS ratio for each of the 15 dermatophyte protein-coding gene. This revealed that all genes are subject to strong negative selection; no statistically significant sites of positive selection were found (see Additional file 1).

**Figure 3:**
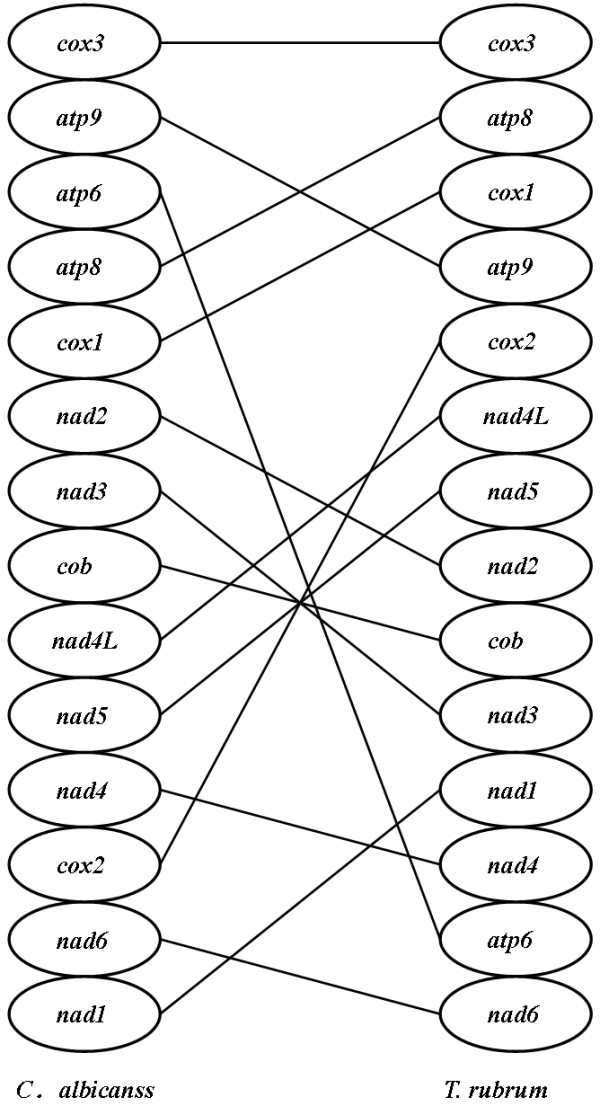
**Comparison of the genomic organization of 14 protein-coding genes in *T. rubrum *and *C. albicans ***(GenBank accession: NC_002653) **mtDNAs**.

The genomic organization of the 15 conserved protein-coding genes in mtDNA is surprisingly identical across all the known dermatophytes (Fig. 2). Furthermore, the same gene order is also largely conserved in other pathogenic filamentous fungi such as *Penicillium marneffei *and *Aspergillus niger *that cause invasive infections [12]. However, the order of these conserved genes is markedly rearranged in the mtDNA of the opportunistic pathogen *Candida albicans *[24] (Fig. 3). The similarity of mtDNA gene order between different fungal species was found to correlate with their evolutionary relationships inferred from phylogenetic analysis (see below).

The large (*rnl*) and small (*rns*) ribosomal RNA genes are present in all dermatophyte mitochondrial genomes at the same relative locations. The *rps5 *gene encodes a ribosomal protein that may play a role in maintaining the integrity of the mitochondrial genome [25]; this gene is present within the intron of *rnl *as expected. The intronic location of this ribosomal protein is therefore maintained in all known dermatophyte mitochondrial genomes.

The previously described *nad4L-nad5 *consecutive gene unit was observed in all 5 mitochondrial genomes. This gene pair is believed to be present in all ascomycota as well as the basidiomycota and zygomycota, but is interrupted by one or more genes in chytridiomycota. This may reflect early phylogenetic divergence of these fungi from the ascomycota [26]. Interestingly, the additional continuous gene unit, *nad4L-nad5-nad2*, characterized by one base overlap and no interruption in gene junctions, is shared by all dermatophyte mitochondrial genomes with the exception of the 2 earlier divergent species, *M. canis *and *T. ajelloi *(see phylogenetic section below). The arrangement of such uninterrupted blocks may reflect strong conservation during evolution from a common ancestor [27]; more dermatophytes may be expected to carry the consecutive *nad4L-nad5-nad2 *gene unit.

### tRNAs and codon usage

All 5 mitochondrial genomes encode the same set of 25 tRNA species with the potential to deliver all 20 amino acids. Multiple tRNA isoacceptors exist for only leucine, serine, arginine and methionine. Interestingly, the organization of the 25 tRNA genes is conserved between all the mitochondrial genomes. The majority of tRNAs (23 of 25) are grouped into 3 clusters flanked by the *atp8-rnl-cox1-atp9 *gene cluster and containing 8, 3 and 12 tRNAs, respectively (Fig. 1). The exceptions are *T. rubrum *and *E. floccosum *where the largest tRNA cluster between *rnl *and *cox1 *is interrupted and subdivided into two sub-clusters by a ~2.5 kb DNA fragment containing additional hypothetical genes (Fig. 2).

tRNA genes have been proposed to play a role in gene shuffling as they are located between protein-coding genes and can act as mobile elements [28]. Homology between isolated tRNA genes may permit genetic recombination, and therefore genetic rearrangement, while gene shuffling is less likely to take place via recombination between conserved clusters of tRNA genes[29,30]. The low number of isolated tRNA genes (2 of 25) may therefore contribute to the high conservation of dermatophyte mtDNA.

All mitochondrial protein-coding genes commence with a classical methionine codon (ATG) and terminate with TAA, the preferred fungal mitochondrial termination codon [31]. The only exception is *atp9 *where TGA is the translation stop codon. Table 2 summarizes codon usages for all protein-coding genes in the 5 mitochondrial genomes. The most frequently used codons are TTA (Leu), ATA (Ile), and TTT (Phe), accounting for about one third of all codons, and indicating a clear preference for amino acids with nonpolar side chains. This is likely to reflect the fact that the majority of the encoded polypeptides are integral membrane proteins. As expected from the AT-rich nature of dermatophyte mtDNA, the third position of the most frequently used codons is strongly biased towards A or T (Table 2). Furthermore, the most rarely-used codons, appearing no more than 5 times in all genomes (TGC, TGG, GGC, CTG, CTC, AGG, CGA, CCG, CCC and ACC), or absent (CGC, CGG, and ACG), all contain 2 or more G/C nucleotides in each codon.

**Table 2:** Codon usage in protein-coding genes of *T. rubrum*, *T. mentagrophytes*, *T. ajelloi*, *M. canis *and *M. nanum*

**Codon**	**AA**	***M. canis***	***M. nanum***	***T. ajelloi***	***T. mentagrophytes***	***T. rubrum***
AAA	Lys (K)	173	158	168	170	209

AAG	Lys (K)	2	9	9	3	8

	Total K	175	167	177	173	217

AAC	Asn (N)	12	20	15	10	17

AAU	Asn (N)	256	259	246	262	307

	Total N	268	279	261	272	324

ACA	Thr (T)	139	129	126	136	146

ACC	Thr (T)	0	0	1	0	0

ACG	Thr (T)	0	0	0	0	0

ACU	Thr (T)	92	100	102	97	101

	Total T	231	229	229	233	247

AGC	Ser (S)	7	6	2	5	6

AGU	Ser (S)	144	149	154	152	165

	Total S	151	155	156	157	171

AUA	Ile (I)	357	353	336	379	394

AUC	Ile (I)	12	18	11	8	19

AUU	Ile (I)	207	209	247	203	231

	Total I	576	580	594	590	644

AUG	Met (M)	114	117	108	116	124

	Total M	114	117	108	116	124

CAA	Gln (Q)	95	94	91	100	105

CAG	Gln (Q)	4	4	4	1	3

	Total Q	99	98	95	101	108

CAC	His (H)	12	9	5	8	8

CAU	His (H)	70	70	77	70	78

	Total H	82	79	82	78	86

CCA	Pro (P)	46	53	38	55	60

CCC	Pro (P)	2	0	1	0	1

CCG	Pro (P)	2	3	0	0	0

CCU	Pro (P)	98	91	106	91	94

	Total P	148	147	145	146	155

CGA	Arg (R)	0	1	3	0	1

CGC	Arg (R)	0	0	0	0	0

CGG	Arg (R)	0	0	0	0	0

AGA	Arg (R)	82	83	86	82	91

AGG	Arg (R)	0	0	0	0	1

CGU	Arg (R)	12	9	9	9	10

	Total R	94	93	98	91	103

CUA	Leu (L)	19	13	16	13	14

CUC	Leu (L)	1	0	0	0	0

CUG	Leu (L)	1	0	1	1	1

UUA	Leu (L)	655	666	658	673	705

UUG	Leu (L)	10	13	12	7	9

CUU	Leu (L)	43	41	53	34	42

	Total L	729	733	740	728	771

GAA	Glu (E)	97	104	105	104	125

GAG	Glu (E)	7	7	4	6	8

	Total E	104	111	109	110	133

GAC	Asp (D)	5	2	5	4	5

GAU	Asp (D)	112	111	108	113	141

	Total D	117	113	113	117	146

GCA	Ala (A)	62	63	63	64	64

GCC	Ala (A)	6	2	2	2	3

GCG	Ala (A)	2	5	0	2	1

GCU	Ala (A)	152	155	156	153	161

	Total A	222	225	221	221	229

GGA	Gly (G)	44	39	36	45	54

GGC	Gly (G)	0	0	1	1	1

GGG	Gly (G)	1	2	1	1	2

GGU	Gly (G)	251	252	253	250	256

	Total G	296	293	291	297	313

GUA	Val (V)	125	124	113	125	140

GUC	Val (V)	1	1	2	1	1

GUG	Val (V)	8	12	6	3	2

GUU	Val (V)	130	122	133	122	124

	Total V	264	259	254	251	267

UAA	Ter (.)	13	13	12	13	14

UAG	Ter (.)	1	1	2	1	2

	Total.	14	14	14	14	16

UAC	Tyr (Y)	13	25	14	18	22

UAU	Tyr (Y)	227	217	229	224	248

	Total Y	240	242	243	242	270

UCA	Ser (S)	148	142	133	148	149

UCC	Ser (S)	2	2	2	1	2

UCG	Ser (S)	0	1	1	1	3

UCU	Ser (S)	127	136	140	133	138

	Total S	277	281	276	283	292

UGA	Trp (W)	61	62	60	61	63

UGG	Trp (W)	1	0	2	1	1

	Total W	62	62	62	62	64

UGC	Cys (C)	1	0	0	0	0

UGU	Cys (C)	31	32	35	30	37

	Total C	32	32	35	30	37

UUU	Phe (F)	339	323	358	346	377

UUC	Phe (F)	65	71	42	54	65

	Total F	404	394	400	400	442

### Intergenic regions and introns

The dermatophyte genomes are highly compact with short intergenic regions. In *M. canis *there is no intergenic region >1 kb, while only one intergenic region exceeds 1 kb in the other genomes. Because protein-coding genes and RNAs are highly conserved between species, the differences in mitochondrial genome sizes between the different dermatophytes are largely explained by length variation in intergenic regions. The longest intergenic region in *M. canis *is only 919 nt while all other intergenic regions in this species are under 500 nt. In contrast, *T. ajelloi *mtDNA contains 4 long intergenic regions (>500 bp) and the longest (located at the *nad2-cob *gene junction) spans >2.5 kb (Fig. 1). This divergence in intergenic regions explains the >4 kb difference in the genome sizes of *T. ajelloi *and *M. canis*.

**Figure 4:**
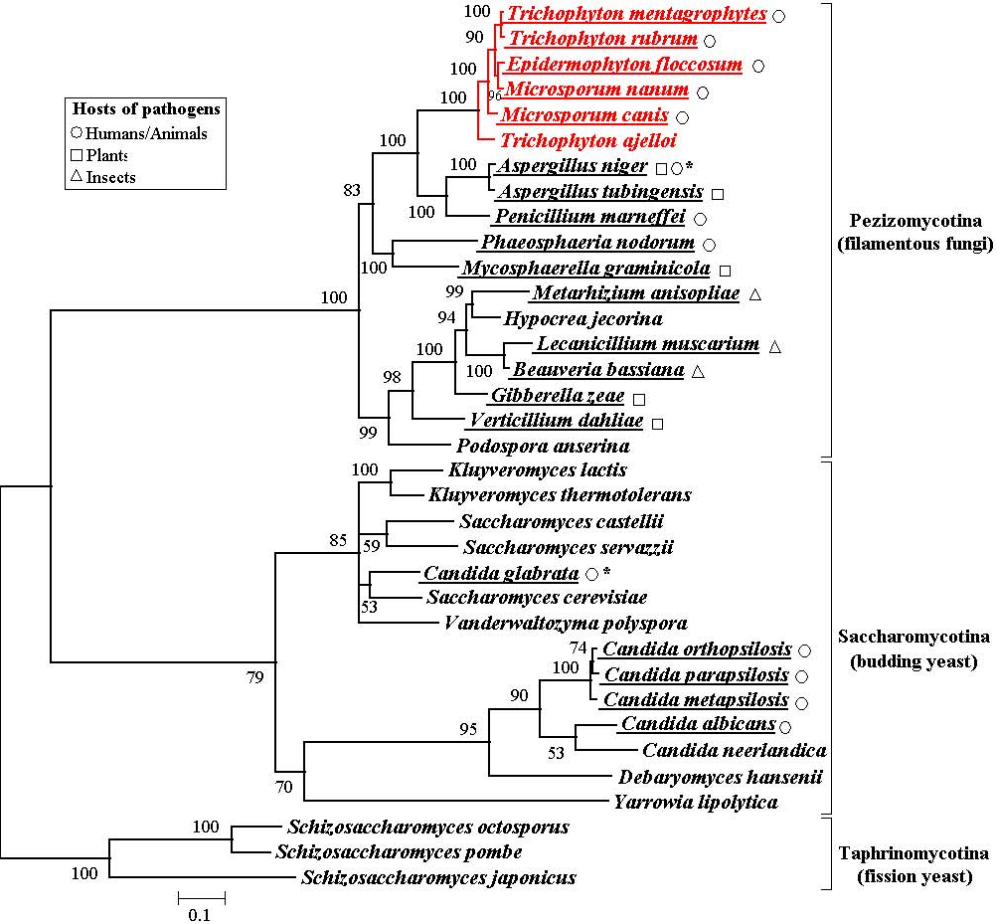
**Maximum likelihood phylogenetic tree based on concatenated mitochondrial proteins**. A total of 4,298 amino acid positions were used for the inference using the TREE-PUZZLE program. Additional sequences obtained from GenBank were: *Epidermophyton floccosum *(AY916130), *Aspergillus niger *(NC_007445), *Aspergillus tubingensis *(NC_007597), *Penicillium marneffei *(NC_005256),*Phaeosphaeria nodorum *(NC_009746), *Mycosphaerella graminicola *(NC_010222), *Metarhizium anisopliae *(NC_008068), *Hypocrea jecorina *(NC_003388), *Lecanicillium muscarium *(NC_004514), *Beauveria bassiana *(NC_010652),*Gibberella zeae *(NC_009493),*Verticillium dahliae *(NC_003060),*Podospora anserina *(NC_001329), *Kluyveromyces lactis *(NC_006077), *Kluyveromyces thermotolerans *(NC_006626), *Saccharomyces castellii *(NC_003920), *Saccharomyces servazzii *(NC_004918), *Candida glabrata *(NC_004691), *Saccharomyces cerevisiae *(NC_001224), *Vanderwaltozyma polyspora *(NC_009638), *Candida orthopsilosis *(NC_006972), *Candida parapsilosis *(NC_005253), *Candida metapsilosis *(NC_006971), *Candida albicans *(NC_002653), *Candida neerlandica *(NC_011133), *Debaryomyces hansenii *(NC_010166), *Yarrowia lipolytica *(NC_002659), *Schizosaccharomyces octosporus *(NC_004312), *Schizosaccharomyces pombe *(NC_001326), *Schizosaccharomyces japonicus *(NC_004332). Bootstrap values obtained in 100 replicates are indicated at the nodes. Dermatophytes are highlighted in red, and pathogenic fungal species are underlined; symbols following indicate their respective hosts (see legend). *A. niger are mainly pathogenic to plants but can infect people if large amounts of spores are inhaled. *C. glabrata *only infect person with lower immunity.

mtDNA intergenic regions diverge not only in size but also in sequence. Indeed, multiple pairwise alignment of the different genomes indicates that colinearities are interrupted almost exclusively in intergenic regions (Fig. 2). However, many intergenic regions were found to be conserved between *T. rubrum *and *T. mentagrophytes*, consistent with the close evolutionary relationship of these 2 species as revealed by phylogenetic analysis (Fig. 4). Remarkably, the longest intergenic region in all genomes is located between *cob *and *nad3 *(with the exception of *T. ajelloi*; see above), but pairwise alignment revealed little similarity between the *cob*-*nad3 *intergenic sequences of the different species as indicated by the blank space in the linear comparison view (Fig. 2). Such divergent intergenic regions may serve as potential genetic markers for species/strain identification of dermatophytes.

Many fungal mitochondrial genomes are intron-rich [32]. In contrast, all the dermatophyte mtDNA gene sequences harbor a single intron (within the *rnl *gene), with the exception of *T. ajelloi *that carries an additional group I intron (801 bp) within *nad1 *(Fig. 1). However, previous mtDNA sequence studies revealed that another strain of *T. rubrum *(IP1817.89) harbors 2 additional introns within *nad1 *and *atp9*, respectively [33]. Moreover, the *E. floccosum *mitochondrial genome carries 4 additional introns located within in *cox1*, *nad5 *(2 introns) and *cob *[12]. The number of introns is therefore strain-specific and also contributes to variation in mitochondrial genome size.

### Phylogenetic analysis

Broad phylogenetic trees of fungi were previously constructed based on rDNA [34-36] or nuclear protein-coding genes [37,38] but these studies did not permit the elucidation of higher-order relationships. A combination of 6 gene regions was recently employed to construct a fungal phylogenetic tree comprising ~200 species [39]. Unfortunately, no dermatophytes were included in this study. We therefore performed phylogenetic analysis based on the complete mitochondrial genomes of 35 species of ascomycota, including 6 dermatophytes, 12 other filamentous fungi, and 17 yeasts (Fig. 4). The high bootstrap values of most nodes indicate the robustness of the tree computed. Fungal species of ascomycota are clustered into 3 distinct groups corresponding to subphyla *Pezizomycotina *(filamentous fungi), *Saccharomycotina *(budding yeast) and *Taphrinomycotina *(fission yeast) respectively (Fig. 4). This confirmed the reliability of mtDNA sequences in fungal phylogenetic analysis.

Interestingly, the tree reported here divides the clade of filamentous fungi into 2 subgroups (Fig. 4). With only a few exceptions, the dermatophytes cluster together with invasive pathogenic fungi of humans and animals, while the other filamentous fungi, mostly pathogens of plants or insects, form a parallel branch (Fig. 4). This suggests that host adaptation has driven the evolution of filamentous fungi. Indeed, previous phylogenetic studies revealed separation between anthropophilic and geophilic species of *Trichophyton *[40] suggesting that ecology is a particularly strong driver of dermatophyte evolution [41].

In the tree established here all the dermatophytes species clustered into a single branch, confirming the monophyletic origin of the dermatophyte lineage. *Aspergillus *[42] and *P. marneffei *[43] comprise a separate branch that shares an immediate ancestor with the dermatophyte group (Fig. 4). However, the 2 sister branches of human pathogenic fungi (causing superficial and invasive infections respectively) are represented by distinct patterns in the phylogenetic tree. The dermatophytes as a group show far less divergence but longer ancestral branch than the *Aspergillus*-*Penicillium *clade (Fig. 4). This indicates the divergence from the latest common ancestor of dermatophytes was later than the *Aspergillus*-*Penicillium *group.

Fossil evidence has allowed dating of the emergence of the ascomycota [44]. Based on this calibration, the dermatophyte lineage may be estimated to have diverged from other fungi at about 32 million years ago (Ma). This result is consistent with a previous rough estimate (~50 Ma) based on nucleotide substitution rates in the small ribosomal subunit RNA [45]. However, the timing of the radiation of the dermatophytes is much later than the divergence of *Candida *and *Saccharomyces *at 723 Ma as previously estimated using 20–188 protein sequences [46]. The high conservation of the dermatophyte mitochondrial genome also suggests that the different dermatophytes diverged only recently.

Conventional phenotypic taxonomy has divided the dermatophytes into 3 genera: *Trichophyton*, *Microsporum *and *Epidermophyton *[47]. Though only a limited number of dermatophyte species were included in the present study, the phylogenetic tree established here does not follow this genus demarcation (Fig. 4). Indeed, recent molecular phylogenetic studies have revealed that both *Trichophyton *and *Microsporum *are paraphyletic [48], prompting reevaluation of the phylogenetic relationships between different dermatophytes [41]. Remarkably, the divergence of *T. ajelloi *from the inferred common ancestor was much earlier than of the other dermatophyte species (Fig. 4). This is consistent with the geophilic features of *T. ajelloi*: the soil environment may have afforded an early ecological niche for all dermatophyte species prior to more recent adaptation to specialized hosts including animals and humans. An earlier study based on 25S rRNA sequences reported that *T. ajelloi *and *T. terrestre *(not included in the present study) are separated from the 'true dermatophyte' [49] and further support the suggestion that *Microsporum*, as well as the zoophilic and anthropophilic *Trichophyton *species, evolved from a geophilic member of *Trichophyton *[48].

## Conclusion

Previous studies into the evolutionary relationships between dermatophyte species have been based on nuclear ribosomal internal transcribed spacers (ITS)[40,50,51], large ribosomal RNA subunits (LSU) [49], chitin synthase (CHS) [52-54]and DNA topoisomerase II genes [55], as well as on PCR fingerprinting [56] and restriction fragment length polymorphism (RFLP) [57] analysis of mitochondrial DNA. The dermatophytes were found to constitute a homogeneous group of species with low genetic diversity contrasting with high phenotypic heterogeneity[41,58]. We now report comparative analysis of 6 complete mitochondrial genomes from all 3 dermatophyte genera (*Trichophyton*, *Microsporum *and *Epidermophyton*). The composition and organization of genes within the mtDNAs of all dermatophytes analyzed was found to be substantially identical, reinforcing the view that dermatophytes are closely related and constitute a highly conserved lineage of filamentous fungi.

Comparative genomics provides a powerful tool for uncovering similarities and differences between species. The present study represents the first application of systematic comparative genomics to dermatophyte phylogeny. The common features shared by all (or the majority) of dermatophyte mitochondrial genomes are as follows. 1. Retention of genome colinearity with high nucleotide sequence similarity (>90%) in coding regions; differences between species are largely restricted to introns and intergenic regions. 2. Strict conservation of the *nad4L-nad5 *gene unit; the consecutive *nad4L-nad5-nad2 *gene unit is also probably present in most dermatophytes. 3. The presence of 3 tRNA gene clusters of identical composition with 2 isolated tRNA genes at identical locations in all dermatophyte mtDNAs. 4. A characteristic rarity of intronic sequences compared to other fungal species.

Phylogenetic analysis has confirmed the monophyletic origin of dermatophytic fungi; these form a distinct clade among filamentous fungi. Compared with other pathogenic fungi such as those causing invasive infections, dermatophytes comprise a closely-related and recently-diverged lineage of ascomycota fungi. The genomic data presented here will allow further exploration of the relationships between different dermatophyte species and will be of general utility in the study of mitochondrial evolution in higher fungi.

## Methods

### Strains and DNA preparation

Type strains of *T. ajelloi *(ATCC 28454),*M. canis *(ATCC 36299), and *M. nanum *(ATCC 42129) and clinical strains of *T. rubrum *(BMU01672) and *T. mentagrophytes *(BMU03104) were kindly provided by Ruoyu Li (Research Center for Medical Mycology, Peking University, Beijing, China). All strains were confirmed by ribosomal ITS sequencing and comparison with the NCBI nucleotide database.

Culture conditions and harvesting of mycelia were as described previously [59]. Mycelia were ground to powder in liquid nitrogen and samples were transferred to liquid nitrogen-cooled 15 ml falcon tubes. Two volumes of lysis buffer 1 (0.35 M sucrose, 50 mM Na_2_EDTA, 20 μg/ml proteinase K, 10 mM Tris.HCl pH 7.4) were added and the mixture were vortexed vigorously for 10 min at 4°C. The lysate was held on ice for 15 min and debris was removed by centrifugation at 3,500 rpm for 10 min at 4°C). Organelles were collected by centrifugation (30,000 rpm, 1 h, 4°C) and resuspended into buffer 2 (150 mM NaCl, 50 mM Na_2_EDTA, 20 μg/ml proteinase K, 10 mM Tris.HCl pH 7.4). Total DNA was isolated using DNeasy Plant Mini Kit (QAIGEN) according to the manufacturer's instructions.

### DNA sequencing, assembly and annotation

Three mtDNA sequences of *T. rubrum *strain IP1817.89 deposited in GenBank (Accession numbers: X65223, X88896 and Y98476) already covered >80% of the mitochondrial genome[60-62]. Four pairs of primers for long-distance and accurate polymerase chain reaction (LA-PCR) were designed according to the known *T. rubrum *sequences and their locations to the complete mitochondrial genome of *E. floccosum *to create a mtDNA sequence scaffold for *T. rubrum *strain BMU016721 (data not shown). The LA-PCR system contained 0.5 μl LATaq polymerase, 5 μl 10 × LA PCR buffer, 8 μl dNTP mixture, 1 μl template DNA, 1 μl primer 1, 1 μl primer 2, and 33.5 μl dH_2_O to give a final volume of 50 μl. LA-PCR conditions were 94°C, 1 min; 98°C, 10 sec; 46.8°C, 15 min; 72°C, 10 min. Steps 2 and 3 were repeated for 30 cycles. All regents were from Takara. LA-PCR products were cloned into the PCR-XL-TOPO vector (Invitrogen). Recombinant plasmids were analyzed by restriction analysis to confirm the presence of the insert DNA. Primer-walking methods were then used to obtain the complete mtDNA sequence of *T. rubrum*.

The complete *T. rubrum *mitochondrial sequence revealed that the genomes were colinear and the overall nucleotide sequence similarity between mtDNAs of *T. rubrum *and *E. floccosum *was >94%, indicating that the mitochondrial genomes of these 2 species are highly conserved. Based on this observation an optimized PCR strategy was devised for mtDNA sequence determination for the other dermatophytes. A selected set of 41 primer pairs used for *T*. rubrum mtDNA sequencing was applied to the sequencing of mtDNAs from the other dermatophyte species. In about half of cases the expected specific amplicons were generated (see Additional file 2). Products of length <2 kb were sequenced after purification with QIAquick Gel Extraction Kit (QAIGEN); larger amplicons were cloned into the PCR-XL-TOPO vector (Invitrogen) prior to sequencing. Further species-specific primers were designed as required for each genome in order to cover remaining sequence gaps; the complete genomes were completed by primer walking.

To confirm the authenticity of the sequences obtained, all 5 genomic sequences were confirmed by overlap PCR covering the complete mtDNA genome with direct sequencing in both directions. All sequencing was performed using an ABI3730 automated sequencer (Applied Biosystems). Sequences were assembled using the Phred/Phrap/Consed package [63,64] with Phred scores set at >20 corresponding to an error rate <1%. The overall sequence quality of each genome was further improved by applying the following 2 criteria to each nucleotide sequenced: coverage by at least 2 independent high-quality (Phred scores >20) reads and a final consensus quality score (Phrap) of >40.

Potential open reading frames (ORFs) were identified using the ORF Finder program  based on genetic code 4. Functional annotation employed BLASTP [64] comparison of translations with the GenBank non-redundant protein database and manual curation. Ribosomal RNA genes were identified by comparison with the published rRNA sequences of *E. floccosum *(GenBank accession: AY916130). Transfer RNA genes were identified using the tRNAscan-SE program [65].

### Comparative genomics and phylogenetic analysis

Genomic comparisons of dermatophyte mtDNAs employed GenomeComp [66]. Orthologs between the mitochondrial genomes of *T. rubrum *and *C. albicans *were identified by bidirectional BLASTP comparisons. Fourteen of the 15 conserved proteins (excluding *Rps5*) were used for whole mitochondrial genome-based phylogenetic analysis of 18 filamentous fungi (including 6 dermatophytes) and 17 yeasts. The sequences of the selected proteins were extracted from the fungal mitochondrial genomes in the GenBank database. Protein sequence alignment was carried out for each individual protein using ClustalW [67]. Multi-alignments were then manually checked and trimmed with BioEdit (version 6.0, by Tom Hall, Department of Microbiology, North Carolina State University, Raleigh). The Datamonkey server was used to calculate the mean dN/dS values of protein-coding genes for dermatophytes [68].

The dataset, a concatenation of 14 proteins comprising 4,298 amino acids, was analyzed by TREE-PUZZLE software [69] to construct the maximum likelihood (ML) tree. Before tree construction, the ProTest software [70] was used to test and determine optimal model-fitting of the sequence data. The WAG model was adopted as optimal selection. The heterogeneity rate was estimated by gamma distribution with 8 rate categories and the α-parameter was estimated from the dataset. Reliability of the dataset was assessed by bootstrap. One hundred permutation datasets were generated using the SEQBOOT program from the PHYLIP package (version 3.68, by Joe Felsenstein, Department of Genome Sciences, University of Washington, Seattle). For each of the 100 datasets a ML tree was constructed using the same parameters as described above. TREE-PUZZLE was then used with the 'consensus of user-defined trees' option to generate a consensus tree. Using the 400 Ma ascomycota fossil [44] as a primary calibration point the dating of dermatophyte divergence was estimated using MEGA 4.0 software [71].

### Data accessibility

The complete mitochondrial genome sequences reported in this paper have been deposited in the GenBank database under accessions: *T. rubrum *(FJ385026), *T. mentagrophytes *(FJ385027), *T. ajelloi *(FJ385028), *M. nanum *(FJ385029) and *M. canis *(FJ385030).

## Authors' contributions

YW performed the culture and isolation of mtDNA from the 5 dermatophytes, primer design and LA-PCR, plasmid construction and sequencing, data analysis, and drafting of the manuscript. JY carried out sequence assembly, genome annotation, comparative and phylogenetic analyses, and revised the manuscript. FY, TL and WL participated in primer design and LA-PCR, plasmid construction and sequencing. YC participated in the design of the Experiment Proposal and revision of the paper. QJ conceived the study, supervised the research and revised the manuscript.

## Supplementary Material

Additional file 1 **Mean dN/dS values of 15 protein-coding genes of 6 dermatophyte species**. This table lists the mean dN/dS values of 15 protein-coding genes of 6 dermatophyte species. Click here for file

Additional file 2 **Forty one primers of *T. rubrum *for LA-PCR test of the other four species and the results**. This table includes primers of *T. rubrum *for LA-PCR test of the other four species and the results. Click here for file
