# Supplementary material for: Recent dermatophyte divergence revealed by comparative and phylogenetic analysis of mitochondrial genomes
Source: BMC Genomics. 2009 May 21;10:238. doi: 10.1186/1471-2164-10-238 (PMC2693141; doi:10.1186/1471-2164-10-238)
Supplement: Additional file 1 — Mean dN/dS values of 15 protein-coding genes of 6 dermatophyte species. This table lists the mean dN/dS values of 15 protein-coding genes of 6 dermatophyte species. [file 1471-2164-10-238-S1.doc]

Table S1. Mean dN/dS values of 15 protein-coding genes of 6 dermatophyte species

*data got at 0.1 significance level

| Genes | Mean dN/dS | Positively selected sites* | Negatively selected sites* |
| --- | --- | --- | --- |
| *atp6* | 0.054 | - | 10 |
| *atp8* | 0.140 | - | - |
| *atp9* | 0.032 | - | - |
| *cob* | 0.190 | - | - |
| *cox1* | 0.024 | - | 19 |
| *cox2* | 0.052 | - | - |
| *cox3* | 0.019 | - | 7 |
| *nad1* | 0.065 | - | 12 |
| *nad2* | 0.088 | - | - |
| *nad3* | 0.027 | - | 6 |
| *nad4* | 0.057 | - | 15 |
| *nad4L* | 0.000 | - | - |
| *nad5* | 0.041 | - | 14 |
| *nad6* | 0.166 | - | - |
| *rps5* | 0.240 | - | 6 |
