# Supplementary material for: Recent dermatophyte divergence revealed by comparative and phylogenetic analysis of mitochondrial genomes
Source: BMC Genomics. 2009 May 21;10:238. doi: 10.1186/1471-2164-10-238 (PMC2693141; doi:10.1186/1471-2164-10-238)
Supplement: Additional file 2 — Forty one primers of T. rubrum for LA-PCR test of the other four species and the results. This table includes primers of T. rubrum for LA-PCR test of the other four species and the results. [file 1471-2164-10-238-S2.doc]

Table S2. Forty one primers of *T. rubrum* for LA-PCR test of the other four species and the results *

| **Primers** | | ***T. mentagrophytes*** | ***T. ajelloi*** | ***M. canis*** | ***M. nunum*** |
| --- | --- | --- | --- | --- | --- |
| 1-F1  1-R1 | CTGCAGTAATATTAGATGGTC | **+** | **-** | **-** | **-** |
|  | AAACCAACAAACGAAGCCTTCCTAA |  |  |  |  |
| 2-F1  2-R1 | TCATGAACATTAACTAAAGAATCA | **+** | **-** | **-** | **+** |
|  | CTACATTAGTTATTATTTTTTGACC |  |  |  |  |
| 3-F1  3-R1 | AGAATTAGGTATAGCTTTTATACAA | **+** | **+** | **+** | **+** |
|  | TAATGTCATACATCAAACTAAATTA |  |  |  |  |
| 4-F1  4-R1 | ATTTTGACTACCTTAAAGAAAGGTT | **+** | **+** | **+** | **+** |
|  | CCATATAGAGCACCTTTTCTATTAC |  |  |  |  |
| 5-F1  5-R1 | CAGGTAGCAGTTAATACGACCTTCT | **+** | **-** | **+** | **+** |
|  | TACCGTTAACCTATAGCCATTTT |  |  |  |  |
| 6-F1  6-R1 | ATAGACGTGCCACATTTTTCATTAC | **+** | **+** | **+** | **-** |
|  | AGATTTGAACTGAAGAGGTGGAG |  |  |  |  |
| 7-F1  7-R1 | ATAGACGTGCCACATTTTTCATTAC | **+** | **+** | **+** | **+** |
|  | CTACATTAGTTATTATTTTTTGACC |  |  |  |  |
| 8-F1  8-R1 | CAGGTAGCAGTTAATACGACCTTCT | **+** | **-** | **+** | **+** |
|  | AAACCAACAAACGAAGCCTTCCTAA |  |  |  |  |
| 9-F1  9-R1 | ATTTTGACTACCTTAAAGAAAGGTT | **+** | **+** | **+** | **+** |
|  | TAATGTCATACATCAAACTAAATTA |  |  |  |  |
| 10-F1  10-R1 | ATGCTATCTTAGGTTTTGCTTTCTC | **+** | **+** | **+** | **+** |
|  | GCAAATGAATGTATAACATCAGCAG |  |  |  |  |
| 11-F1  11-R1 | TTTACCTGTGCAATCTTAAGTTTTT | **-** | **-** | **+** | **+** |
|  | GTAAATAAAAAAAAAAAGTATCAAT |  |  |  |  |
| 12-F1  12-R1 | TTTACCTGTGCAATCTTAAGTTTTT | **+** | **-** | **+** | **+** |
|  | TAAAGTATGGAGCTAATGAAAA |  |  |  |  |
| 13-F1  13-R1 | TACTTTCTATGGGAGCTGTTTTTGC | **+** | **+** | **-** | **+** |
|  | GCAAATGAATGTATAACATCAGCAG |  |  |  |  |
| 14-F1  14-R1 | TGAATTAGCTGGTGGTGGTGATCCT | **+** | **-** | **-** | **+** |
|  | ACAAAACTTTTTATAATAGTATTCA |  |  |  |  |
| 15-F1  15-R1 | AAATGGTGCAGGTACAGGTTGAACA | **-** | **-** | **-** | **-** |
|  | GTAAATAAAAAAAAAAAGTATCAAT |  |  |  |  |
| 16-F1  16-R1 | TTCAGGTTTGGTTGGTACAGCTTTC | **-** | **-** | **-** | **-** |
|  | GTAAATAAAAAAAAAAAGTATCAAT |  |  |  |  |
| 17-F1  17-R1 | TTTTGGTATTTAGCTATGAGTGTT | **+** | **-** | **-** | **-** |
|  | GTAAATAAAAAAAAAAAGTATCAAT |  |  |  |  |
| 18-F1  18-R1 | TTAGGTGTAGAAGGTGTAGGAGTT | **+** | **-** | **+** | **+** |
|  | TTAAATAAAGTTGGAAGTGC |  |  |  |  |
| 19-F1  19-R1 | TTTTTCTATATTCTTTGGTTA | **+** | **+** | **+** | **+** |
|  | ATTGTATATTGTTCTCCTT |  |  |  |  |
| 20-F1  20-R1 | TTGTCTTAAAATTAGGTGGTCA | **+** | **-** | **+** | **+** |
|  | TAAGATTAGTTGGTTGTGGTGA |  |  |  |  |
| 21-F1 | CAGATGTTTATGATGGTATTCC | **+** | **-** | **-** | **-** |
| 21-R1 | ATAACCAGATGCTACAGTCAG |  |  |  |  |
| 22-F1  22-R1 | TTTAGTAGAATTTATTTGAGGAG | **+** | **+** | **-** | **-** |
|  | ATAACCAGATGCTACAGTCAG |  |  |  |  |
| 23-F1  23-R1 | TTTAGTAGAATTTATTTGAGGAG | **+** | **+** | **-** | **+** |
|  | TAACCACCTAAAAATAAAACACTG |  |  |  |  |
| 24-F1  24-R1 | TGGGTCAAAATAGAACACAG | **+** | **+** | **+** | **+** |
|  | TAATAAAAATAAAGGTGGTAA |  |  |  |  |
| 25-F1  25-R1 | TAACAACAATAATAGAAAGTCAA | **+** | **-** | **+** | **+** |
|  | TAATAAAAATAAAGGTGGTAA |  |  |  |  |
| 26-F1  26-R1 | TAGCAATAAAAACAGCTTTCCTTAT | **+** | **+** | **+** | **+** |
|  | TACACAAATAAATAAACCAGATGA |  |  |  |  |
| 27-F1  27-R1 | TAATAGCTTATTCATCGGTTTCTCA | **+** | **+** | **+** | **+** |
|  | ATAAAAAGTGATGTTGAAGAAAAAA |  |  |  |  |
| 28-F1  28-R1 | ATGCTATCTTAGGTTTTGCTTTCTC | **+** | **+** | **+** | **+** |
|  | AATGGTTTTAATCCACCATATTTTC |  |  |  |  |
| 29-F1  29-R1 | CAGGTAGCAGTTAATACGACCTTCT | **-** | **-** | **+** | **+** |
|  | CCATATAGAGCACCTTTTCTATTAC |  |  |  |  |
| 30-F1  30-R1 | GATTCGATTTCCCATGACTCCTATA | **+** | **+** | **+** | **+** |
|  | TACACAAATAAATAAACCAGATGA |  |  |  |  |
| 31-F1  31-R1 | TTAGGTGTAGAAGGTGTAGGAGTT | **-** | **+** | **-** | **-** |
|  | GGGGATGTACTAAATGTAATGAAAT |  |  |  |  |
| 32-F1  32-R1 | TTCTGTATAATTTTTTGTTGTGCTA | **-** | **-** | **-** | **-** |
|  | TAACCACCTAAAAATAAAACACTG |  |  |  |  |
| 33-F1  33-R1 | CTGCAGTAATATTAGATGGTC | **+** | **-** | **-** | **-** |
|  | AGATTTGAACTGAAGAGGTGGAG |  |  |  |  |
| 34-F1  34-R1 | TTTTGGTATTTAGCTATGAGTGTT | **+** | **+** | **+** | **+** |
|  | TAAAGTATGGAGCTAATGAAAA |  |  |  |  |
| 35-F1  35-R1 | AGGAGCACAATTAATAACATCAAG | **+** | **-** | **-** | **-** |
|  | ACAGCAGGTACTACAAGACTAAT |  |  |  |  |
| 36-F1  36-R1 | TACCATTAAACTACTCGGGCTAATT | **+** | **+** | **-** | **-** |
|  | TTGGTAGTGCTATTAATCTTTCTG |  |  |  |  |
| 37-F1  37-R1 | AGCCCCTACAACTGTCCCTACT | **-** | **-** | **-** | **-** |
|  | CGGCTGGGTAGGCACCTCATTTGGG |  |  |  |  |
| 38-F1  38-R1 | ATCATCTGGAGGTCCTTTTACTTC | **-** | **-** | **-** | **-** |
|  | CTTTTTATACGGATTTAGGATTC |  |  |  |  |
| 39-F1  39-R1 | TAGCCATAAAAAAAGGGTTAGTAAC | **+** | **+** | **+** | **+** |
|  | ATACTCTACTTGCTTACTTGCGATA |  |  |  |  |
| 40-F1  40-R1 | GGTCAACATTCCCAACTCCTT | **+** | **+** | **+** | **+** |
|  | AAGTTCATATTGTAATTTTGTAGA |  |  |  |  |
| 41-F1  41-R1 | TTATTTAATTAGGTTTTGATGAA | **+** | **-** | **+** | **+** |
|  | ACGCGACTGATAGACTTGTAA |  |  |  |  |

* + produced expected amplicons; - No products.
